# *N*-Acetylcysteine overcomes epalrestat-mediated increase of toxic 4-hydroxy-2-nonenal and potentiates the anti-arthritic effect of epalrestat in AIA model

**DOI:** 10.7150/ijbs.85028

**Published:** 2023-08-06

**Authors:** Linna Wang, Baixiong Huang, Yaling Zeng, Jiujie Yang, Zhi Li, Jerome P. L. Ng, Xiongfei Xu, Lu Su, Xiaoyun Yun, Liqun Qu, Ruihong Chen, Weidan Luo, Yuping Wang, Chang Chen, Lijun Yang, Yuanqing Qu, Wei Zhang, Joyce Tsz Wai Chan, Xingxia Wang, Betty Yuen Kwan Law, Simon Wing Fai Mok, Sookja Kim Chung, Vincent Kam Wai Wong

**Affiliations:** 1 Dr. Neher's Biophysics Laboratory for Innovative Drug Discovery, State Key Laboratory of Quality Research in Chinese Medicine, Macau University of Science and Technology, Macau, China.; 2 Faculty of Medicine, Macau University of Science and Technology, Macau, China.; 3 Centro Hospitalar Conde de São Januário, Macau, China.; 4 State Key Laboratory of Pharmaceutical Biotechnology, The University of Hong Kong, Hong Kong, China.; 5 Macau Medical Science and Technology Research Association, Macau, China.

**Keywords:** Epalrestat, AR inhibitor, Rheumatoid arthritis, 4-hydroxynonenal, *N*-acetylcysteine

## Abstract

Epalrestat, an aldose reductase inhibitor (ARI), has been clinically adopted in treating diabetic neuropathy in China and Japan. Apart from the involvement in diabetic complications, AR has been implicated in inflammation. Here, we seek to investigate the feasibility of clinically approved ARI, epalrestat, for the treatment of rheumatoid arthritis (RA).

The mRNA level of AR was markedly upregulated in the peripheral blood mononuclear cells (PBMCs) of RA patients when compared to those of healthy donors. Besides, the disease activity of RA patients is positively correlated with AR expression. Epalrestat significantly suppressed lipopolysaccharide (LPS) induced TNF-α, IL-1β, and IL-6 in the human RA fibroblast-like synoviocytes (RAFLSs). Unexpectedly, epalrestat treatment alone markedly exaggerated the disease severity in adjuvant induced arthritic (AIA) rats with elevated Th17 cell proportion and increased inflammatory markers, probably resulting from the increased levels of 4-hydroxy-2-nonenal (4-HNE) and malondialdehyde (MDA). Interestingly, the combined treatment of epalrestat with *N*-Acetylcysteine (NAC), an anti-oxidant, to AIA rats dramatically suppressed the production of 4-HNE, MDA and inflammatory cytokines, and significantly improved the arthritic condition.

Taken together, the anti-arthritic effect of epalrestat was diminished or even overridden by the excessive accumulation of toxic 4-HNE or other reactive aldehydes in AIA rats due to AR inhibition. Co-treatment with NAC significantly reversed epalrestat-induced upregulation of 4-HNE level and potentiated the anti-arthritic effect of epalrestat, suggesting that the combined therapy of epalrestat with NAC may sever as a potential approach in treating RA. Importantly, it could be regarded as a safe intervention for RA patients who need epalrestat for the treatment of diabetic complications.

## Introduction

Rheumatoid arthritis (RA) is a chronic and progressive autoimmune disease, with 0.5-1% global incidence and an estimated 5 million patients in mainland China 1. The major pathological characteristics of RA are chronic synovitis, the formation of pannus and erosion of articular cartilage and bones, ultimately leading to deformity 2. In the development of RA, the infiltration of immune cells into the articular joints, generation of reactive oxygen species (ROS), and production of a variety of pro-inflammatory cytokines 3, 4. Rheumatoid arthritis synovial fibroblasts (RASFs) also have been extensively implicated in the pathogenesis of RA *via* secretion of pro-inflammatory factor and damage of articular 5. As such, interventional strategies targeting inflammation and oxidative stress represent effective routes for manipulating the development of RA. Currently, conventional disease-modifying antirheumatic drugs (DMARDs), especially methotrexate (MTX), are the mainstays of medical treatment against RA 6-8. However, the rate of disease remission remains unsatisfactory, and the long-term treatment of MTX leads to drug resistance and adverse side effects, such as cardiovascular complications, liver and kidney damages 9-14. Moreover, biological therapies, such as rituximab and tocilizumab, for the treatment of RA are unaffordable for most patients 15. Besides, the ineffectiveness of individual biological therapies has been reported in ~40% patients 16. Therefore, exploring novel therapeutic approaches that can effectively repress the inflammatory responses with low toxicity is undoubtedly needed for ensuring the quality of life in RA patients.

Aldose reductase (AR) is the first and rate-limiting enzyme of polyol pathway responsible for the reduction of glucose to sorbitol, which is further metabolized to fructose by sorbitol dehydrogenase under hyperglycemic status. AR has been implicated in the etiology of secondary complications of diabetes, and AR inhibitor (ARI) epalrestat has been widely adopted in clinical management of secondary diabetic complications in mainland China 17 and Japan 18. In addition, AR is also closely implicated in inflammation. Some scientists believed that one of the mechanisms by which ARI generates beneficial effects in diabetic complications may be inhibition of inflammation 19. The reduced product of the glutathione (GS)-lipid aldehyde conjugate catalyzed by AR is an important inducer to activate PLC/PKC/MAPK, AP-1 and NF-κB signaling cascades 20, 21, which are known to participate in many inflammation related diseases, including RA. Accumulating evidences suggest that inhibition of AR can suppress immune and inflammatory responses in different diseases 22-24. For instance, engelietin was demonstrated to suppress inflammation through inhibiting AR-dependent activation of NF-κB and MAPK in pelvic inflammatory disease 25. Sorbinil, an AR inhibitor, was also shown to inhibit the polymicrobial sepsis-mediated release of MCP-1, IL-1β, IL-6, TNF-α 26. Of note, clinically used AR inhibitor, epalrestat exhibit an anti-inflammatory effect in the management of diabetic complications 17. Furthermore, inhibition of AR has been reported to suppress ragweed pollen extract-induced airway inflammation 27. The depletion of AR attenuated ischemia-reperfusion injury through blockage of the infiltration of neutrophil and macrophage, down-regulation of pro-inflammatory cytokines/chemokines, and inactivation of JNK and NF-κB pathways 28. Owing to the close relationship between RA activity with inflammation, and in accordance with the anti-inflammatory property of ARI, it is tempted to propose that the inhibition of AR activity could be a potential strategy in the treatment of RA. Amongst the different ARIs, epalrestat appeared as the most ideal repurposing candidate for RA due to its long history of efficacy and safety in clinical use against diabetic neuropathy (DN) 29-33.

On the other hand, AR can also serve as an anti-oxidant enzyme that helps removing the metabolites generated from lipid peroxidation, including hydroxyl radical, hydrogen peroxide, etc. 34. AR is usually upregulated in the area of high 4-hydroxy-2-nonenal (4-HNE) formation to reduce both free aldehydes and their glutathione conjugates, particularly under oxidative stress arising from overwhelming of other antioxidant mechanisms 19. Several studies indicated that the inhibition of AR causes the accumulation of toxic aldehydes, such as 4-HNE 35-37, the most abundant lipid peroxidation products, also one of the substrates of AR 38. These highly reactive aldehydes are reported to contribute to persistent oxidative stress-related diseases including RA 39. It could be engaged in multi-step regulation of cellular pathways through modifying proteins involved in cellular homeostasis 40, 41. Furthermore, aldehydes can increase adhesion of monocyte, promote production of pro-inflammatory cytokines and trigger inflammation 42, 43. A recent review summarized that 4-HNE-protein adducts may accelerate inflammation, even acts as autoantigens in the vicious circle of autoimmune diseases 44. Notably, increased levels of 4-HNE and its adducts have frequently been observed in RA patients 40, 45. In arthritic condition, AR-mediated detoxification of lipid peroxidation products seems to contradict with the anti-inflammatory effect of AR inhibition. Notably, the prevalence of insulin resistance (IR) in RA patients is about 40%, which predicts the development of type 2 diabetes (T2D) 46. Consistently, RA patients have a nearly 1.5 times higher risk of developing T2D than general population 47. Furthermore, it has been reported that the prevalence of diabetes mellitus in patients with RA is around 15% to 19%, significantly higher than the normal population 47-50. These findings suggested that RA patients who also have diabetes may need epalrestat for the management of diabetic complications. Nevertheless, the specific role of AR in or the effect of AR inhibitors, epalrestat, on RA pathogenesis is still not clear. Thus, it is imperative to examine the role of AR in RA and whether the use of ARI could influence the development of RA.

In the present study, we illustrated that AR is upregulated in RA patients and AIA model, and is involved in the reduction of 4-HNE. We confirmed that the inhibition of AR by epalrestat alone lead to more severe RA-like phenotype in AIA rats due to the accumulation of 4-HNE and aggravated autoimmune responses, such as T cell activation and release of proinflammatory cytokines. Of note, *N*-acetylcysteine (NAC) is a well-tolerated and safe medicine that exhibits antioxidant properties by scavenging ROS and activating endogenous defense system against oxidant injury 51. Co-treatment with NAC significantly reduced the accumulation of toxic 4-HNE, and exhibited combined anti-arthritic effect with decreased arthritic score and reduced inflammatory markers in AIA rats. Accordingly, AR inhibitor epalrestat should be prescribed with great attention for the diabetic patients with RA or other autoimmune diseases. Anti-oxidants like NAC are strongly recommended for the removal of toxic aldehydes including 4-HNE in RA patients. Besides, the combined therapy of epalrestat and NAC may be considered as a potential and safe therapeutic option for RA patients with diabetic complications.

## Results

### RA patients and AIA rats show increased expression of AR

**Figure 1:**
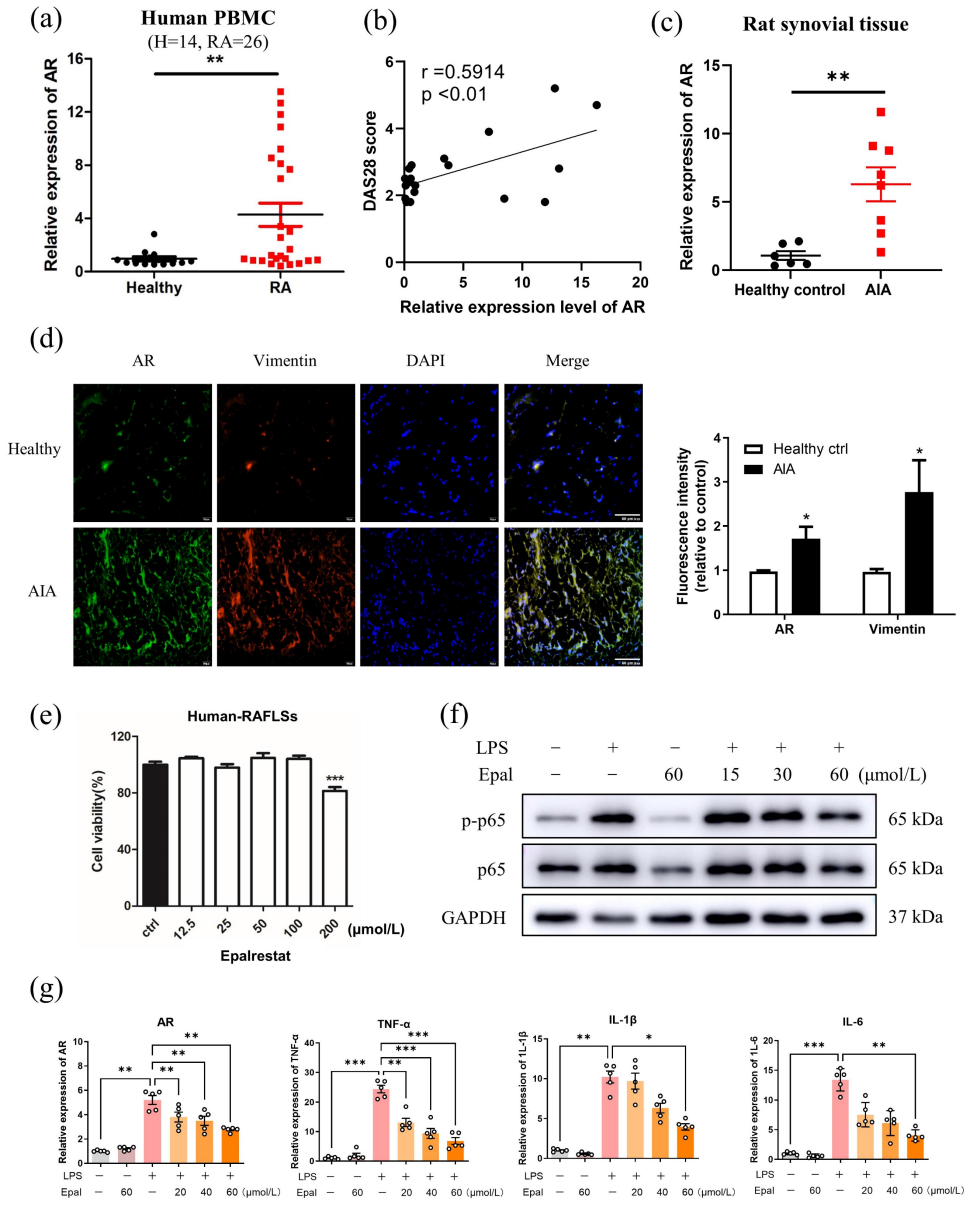
** Expression and functional role of AR in rheumatoid arthritis. (a)** Gene expression level of AR in PBMCs from healthy volunteers (n = 14) and patients with RA (n = 26). **(b)** The correlation between mRNA level of AR and the disease activity of RA patients, r=0.5914, p<0.01. **(c)** Gene expression level of AR in rat synovial tissue from healthy controls (n = 6) versus AIA rat models (n=8). **(d)** Immunofluorescence staining of AR in synovial tissue from healthy control rats and AIA rats. Synovium tissues were sectioned and immune-stained with DAPI (nuclear staining) and antibodies against AR and vimentin prior to secondary antibody treatment (FITC anti-mouse 2^nd^ antibody for AR; TRITC anti-rabbit 2^nd^ antibody for vimentin), scale bar: 60 μm. Bar charts represent the *Image J* quantitation of the fluorescence intensity of AR and vimentin in synovium tissues relative to the expression in healthy controls. **(e)** Cell cytotoxicity of epalrestat in RAFLS. RAFLS were treated with indicated concentrations of epalrestat for 24 hrs. **(f)** Effect of epalrestat on LPS induced phosphorylation of p65.** (g)** Epalrestat suppressed LPS-induced gene expression of AR and pro-inflammatory cytokines. RAFLS were pre-treated with DMSO or 15 - 60 μmol/L of epalrestat for 2 hrs with or without stimulation with 150 ng/mL LPS for 4 hrs. RAFLS were then harvested for Western blot or RT-PCR. The data shown are the means ± SEM (n ≥3). *P<0.05, **P<0.01, ***P<0.001 *vs* untreated or LPS-treated RAFLS.

Several studies have shown that AR is markedly upregulated in a number of cancers 52. In inflammation-related diseases, high expression of AR was usually observed in immune cells, including macrophages and T cells 35. To investigate whether AR expression associated with the inflammatory condition of RA, firstly, the mRNA expression level of AR in PBMCs of RA patients and healthy volunteers were assessed. As depicted in Fig. 1a, the gene expression level of AR in RA patients was significantly higher than that of healthy individuals. Disease Activity Score (DAS28) was also confirmed with the positive correlation between gene expression of AR and disease severity of RA with r =0.5914, p<0.01 (Fig. 1b). To determine the potential role of AR in RA, adjuvant induced arthritic (AIA) model was used since it develops chronic synovitis, which is the most typical characteristics of RA and is a well-accepted experimental model for studying RA. Similarly, the mRNA level of AR was significantly increased in the synovium of AIA rats compared to healthy controls (Fig. 1c). Notably, immunofluorescence staining using the antibody against AR further showed the increased trend of AR (green) expression along with the increased level of vimentin (red) in the synovial tissues of AIA rats (Fig. 1d). Vimentin was used as a marker to indicate the progress of RA 53. These results suggested that AR expression was elevated in the PBMCs of RA patients and synovium of AIA rats, implying that AR may be closely associated with the pathogenesis of RA.

Rheumatoid arthritis fibroblast-like synoviocytes (RAFLSs) originate from RASFs which have been extensively implicated in RA pathogenesis and are a well-adopted cell model for RA research. We next examined the anti-inflammatory effect of epalrestat in RAFLSs, and found that epalrestat inhibited the LPS-mediated phosphorylation of p65, and suppressed gene expression of AR, TNF-α, IL-1β and IL-6 in a concentration-dependent manner without affecting cell viability (Fig. 1e-g). These findings indicated that the expression level of AR increases with inflammation, and inhibition of AR by epalrestat effectively suppressed the inflammatory response through inactivation of NF-κB pathway *in vitro*.

### AR inhibitor epalrestat exacerbates the disease severity in AIA rats

**Figure 2:**
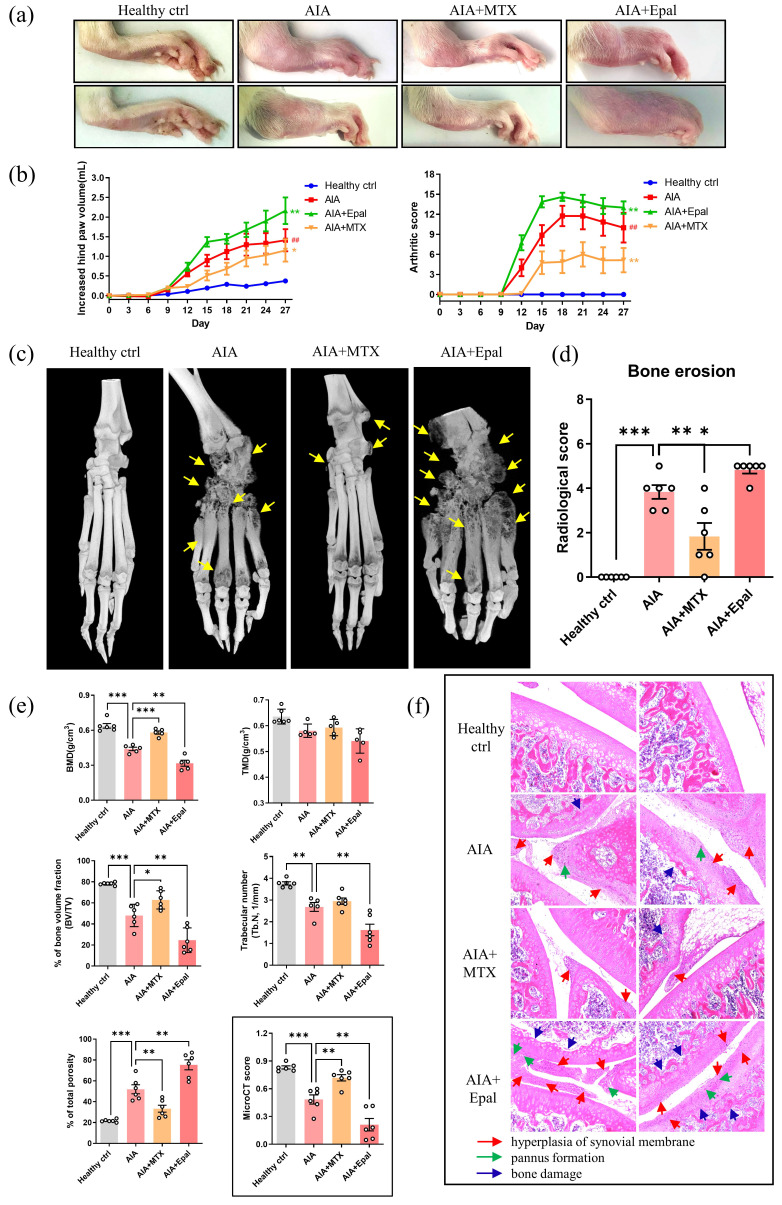
** AR inhibitor, epalrestat exacerbates the disease severity in AIA rats. (a)** Representative images of hind paw swelling from AIA rats after epalrestat treatment. Hind paw swelling images were captured on Day 27.** (b)** The hind paw swelling and arthritis scores of epalrestat-treated AIA rats. Three groups of AIA rats were treated with vehicle, positive control drug methotrexate (MTX, 7.6 mg/kg/week) and epalrestat (Epal, 15.5 mg/kg/day) after arthritis induction for total 27 days, hind paw volumes (ml) and arthritis scores were evaluated every 3 days. **(c)** Representative micro-CT radiographic images of damaged hind limb joint bone. Yellow arrows indicate the severity of bone erosion.** (d)** Radiological scores of epalrestat-treated AIA rats. The radiological scores were obtained according to the severity of bone erosion shown in micro-CT images. **(e)** The micro-CT scores and five disease-related index of micro-CT analyses of the calcaneus-bone mineral density (BMD), bone volume fraction (BV/TV), cortical mineral density (g/cm^3^) (TMD), trabecular number (mm^-1^) (Tb. N), and the percentage of total porosity of epalrestat-treated AIA rats. The data are expressed as the means ± SEM (n ≥5). *P<0.05, **P<0.01, ***P<0.001, significantly different compared with healthy control or vehicle control. **(f)** Representative histological images of knee joints stained with H & E in epalrestat treated AIA rats (Magnification, ×100). Blue arrows indicate bone loss; green arrows indicate the pannus formation; red arrows indicate hyperplasia of synovial membrane.

Following the promising results obtained from the use of epalrestat in suppressing inflammation in RAFLS, we then assessed the anti-arthritic effects of epalrestat in AIA rats. Joint rigidity, swelling and erythema were identified in the paw of AIA rats when compared to healthy controls. Surprisingly, epalrestat treated AIA rats displayed more severe phenotype of arthritis in the paw, and markedly increase in the hind paw volume and arthritic score during day 9-18 compared to AIA model (Fig. 2a and 2b). The hind paw swelling was first observable on day 8 in epalrestat-treated AIA rats, which was earlier than AIA model (Data not shown). The microCT images displayed mutilated bone loss in epalrestat treated AIA rats (Fig. 2c). Analysis of bone mineral density (BMD), cortical mineral density (TMD), bone volume fraction (bv/tv), trabecular number (Tb.N) and total porosity further verified that epalrestat treatment caused more severe bone destruction compared to the vehicle-treated AIA rats. The overall radiological scores and microCT scores also suggested significant severe bone destruction in epalrestat-treated AIA rats when compared to AIA rats (Fig. 2d and 2e). Besides, hematoxylin and eosin (H&E) staining showed more severe arthritic characteristics of joints with synoviocytes hyperplasia with infiltration of immune cells and plasma cells, cartilage loss, interstitial fibroblast proliferation accompanied by neovascularization in epalrestat-treated AIA rats (Fig. 2f). Collectively, these results demonstrated that epalrestat treatment aggravated the arthritic condition of AIA rats.

### Epalrestat promotes inflammatory condition of AIA rats *via* increasing Th17 cell populations

**Figure 3:**
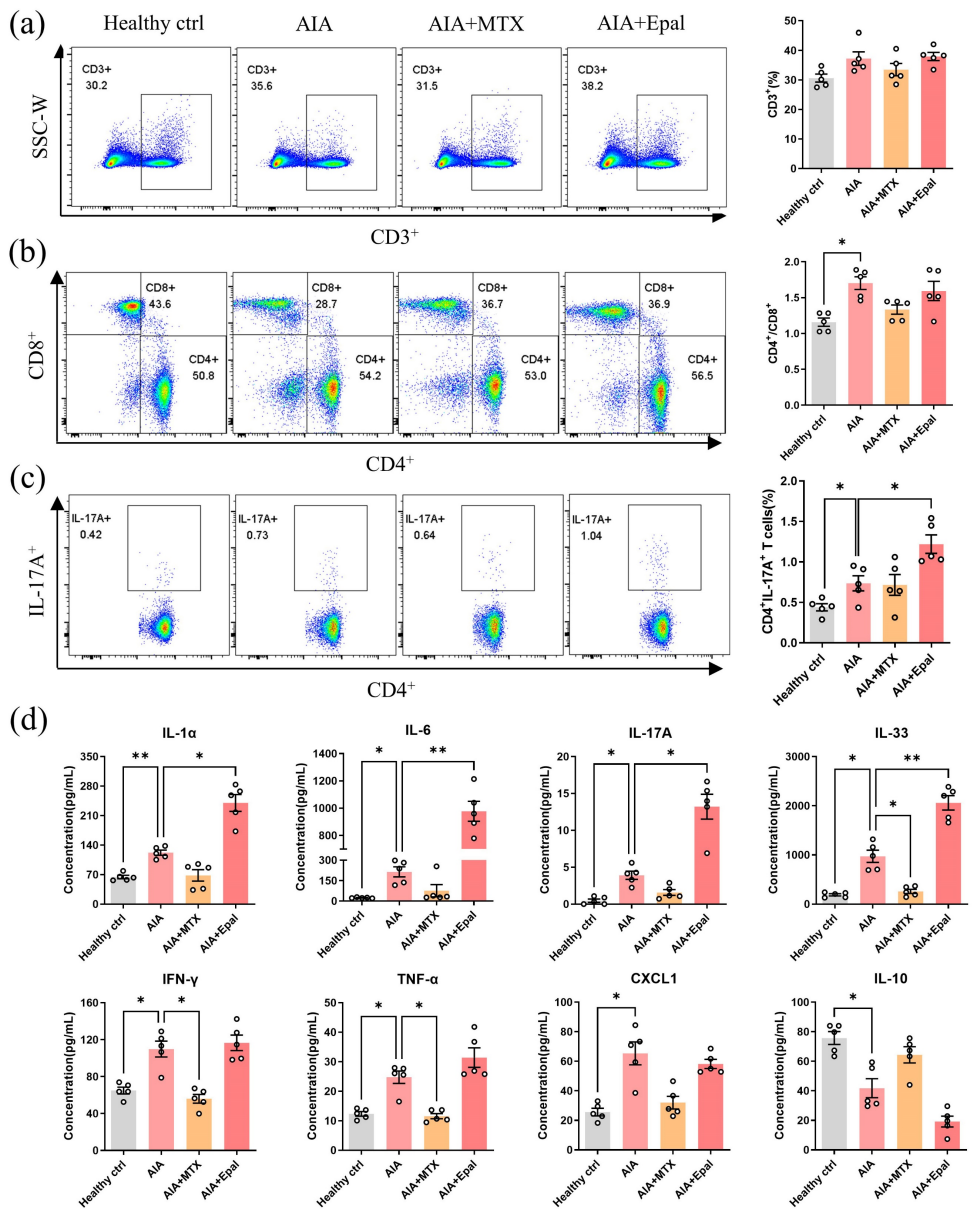
** Epalrestat enhances autoimmune response in AIA rats.** Blood lymphocytes were harvested from epalrestat-treated AIA rats for flow cytometry analysis using fluorescent antibodies against CD45, CD3, CD4, CD8 and IL-17A. **(a)** Representative flow cytometry images show the percentage of CD3^+^ T lymphocytes gated on CD45^+^ lymphocytes. The bar chart shows the proportion of CD3^+^ T cells among CD45^+^ lymphocytes. **(b)** Representative flow cytometry images show the proportion of CD4^+^ T cells and CD8^+^ T cells gated on CD3^+^ T lymphocytes. The quantitative bar chart shows the ratio of CD4^+^ / CD8^+^ T cells. **(c)** Representative flow cytometry images show the percentage of the IL-17A^+^ T cells gated on CD4^+^ T lymphocytes. The bar chart shows the percentage of IL-17A^+^ T cells among CD4^+^ T cells. **(d)** The serum concentration of inflammatory cytokines in epalrestat-treated AIA rats. The concentration of these cytokines was measured using LEGENDplex^TM^ Rat Inflammatory Panel. The data are presented as the means ± SEM (n ≥5). *P<0.05, **P<0.01, ***P<0.001, significant different compared with healthy control or vehicle control.

Multiple immune cells types misfunction plays a key pathogenic role in RA development 54. Aberrant activation of Th (T helper) 17 cells is considered to be a pivotal manifestation in the pathogenesis of autoimmune disease including RA 55. The increased differentiation of Th17 cells promotes the secretions of IL-17A, IL-22, IL-26 and subsequently induces the production of pro-inflammatory cytokines, such as TNF-α, IL-6 and IL-1β 56. Assessment of T cells in peripheral blood lymphocytes showed that the proportion of CD3^+^ T cell and the ratio of CD4^+^/CD8^+^ were increased in AIA rats, and epalrestat treatment didn't change it markedly compared to vehicle- treated AIA rats (Fig 3a and 3b). Strikingly, increased proportion of IL-17A-producing CD4^+^ T cells (Th17 cells) was observed in AIA rats compared with healthy controls, this frequency was markedly increased in epalrestat treated AIA rats (Fig 3c). The increased serum concentration of IL-17A further confirmed the abnormally increased differentiation of Th17 cells after epalrestat treatment (Fig 3d). Furthermore, we also detected the serum concentration of inflammation related cytokines in epalrestat treated AIA rats. As shown in Fig. 3d, the concentration of pro-inflammatory cytokines was dramatically up-regulated in AIA model group, in particular, the concentration of IL-1α, IL-6, IL-17A, IL-33 were much higher in epalrestat treated AIA rats in comparison with vehicle treated AIA rats. Whereas, the concentration of IL-10 in AIA rats was significantly down-regulated, and was even lower upon epalrestat treatment (Fig. 3d), which is an anti-inflammatory cytokine produced by Treg cells, and inhibits the secretion of some pro-inflammatory cytokines 57. Accordingly, these results illustrated that the inhibition of AR by epalrestat increased the Th17 cell proportion and facilitated the inflammatory response *in vivo* in spite of its anti-inflammation effect *in vitro*.

### Epalrestat promotes oxidative stress and causes the accumulation of 4-HNE in AIA rats

**Figure 4:**
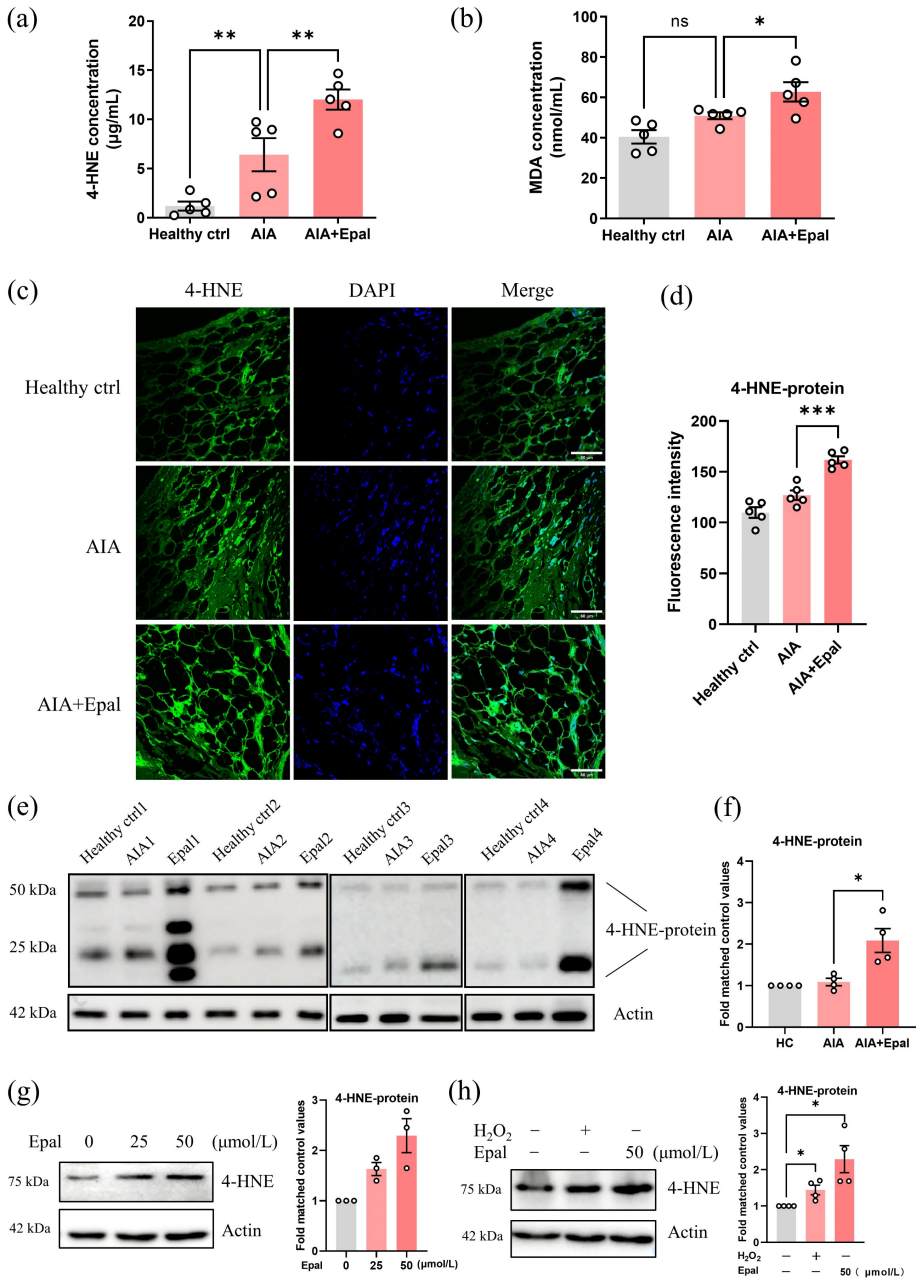
** Epalrestat treatment facilitates the accumulation of 4-HNE in AIA rats. (a)** The changes of the 4-HNE concentration in the serum of AIA rats with epalrestat treatment. **(b)** The changes of the MDA concentration in the serum of AIA rats with epalrestat treatment.** (c)** Immunofluorescence staining of 4-HNE-protein conjugate in the knee synovial tissue of AIA rats with epalrestat treatment. Synovium tissues were sectioned and immune-stained with antibodies against 4-HNE-protein conjugate prior to secondary antibody (FITC) treatment, scale bar: 60 μm. **(d)** Bar charts show the quantitation of the fluorescence intensity of 4-HNE-protein conjugate in the synovium tissues using* Image J*. **(e)** Expression level of 4-HNE-protein conjugate in epalrestat treated AIA rats. Synovial samples were harvested for extraction of protein and Western blotting using antibodies against 4-HNE-protein conjugate and β-actin (as the loading control). **(f)** Histogram show the quantitation of 4-HNE-protein conjugate expression relative to the healthy controls. **(g&h)** Epalrestat dose-dependently induced the expression of 4-HNE-protein conjugate in RAFLS. Cells were treated with 25-50 μmol/L epalrestat overnight or 500 μmol/L H_2_O_2_ for 4 hrs. Cell lysates were analyzed by Western blotting using antibodies against 4-HNE-protein conjugate and β-actin (loading control). The data are presented as the means ± SEM (n ≥ 3). *P<0.05, **P<0.01, significant difference when compared to AIA group.

Since inhibition of AR by epalrestat exaggerated the immune response in AIA rats, we postulated that the upregulation of AR in RA condition may be responsible for the detoxification of toxic aldehydes especially 4-HNE. Inhibition of AR may lead to the accumulation of these aldehydes and exaggerate autoimmune responses, thus accelerate the development of RA. Therefore, we next detected the serum content of 4-HNE in epalrestat-treated AIA rats as well as the oxidative stress marker MDA. Compared to healthy controls, the serum concentration of 4-HNE was significantly upregulated in AIA rats, and the content of 4-HNE was much higher in epalrestat-treated group than AIA group (Fig. 4a). Notably, the concentration of MDA was only slightly increased in AIA rats when compared with healthy controls (no statistical significance), and was increased to a greater extent after epalrestat treatment (Fig. 4b). Besides, immunofluorescent staining showed that the treatment of epalrestat increased 4-HNE-protein conjugate level in synovial tissue compared to that of vehicle-treated AIA rats (Fig 4c and 4d). Western blotting analysis further confirmed the obvious elevation of 4-HNE-protein conjugate in the synovial tissue of epalrestat-treated AIA rats (Fig 4e and 4f). As the most abundant lipid peroxidative product, 4-HNE could be induced by a strong oxidant, H_2_O_2_ (Fig. S1a). Consistently, *in vitro* study verified that epalrestat treatment increased 4-HNE protein conjugate level in a dose dependent manner in RAFLS (Fig. 4g), and this increment was much higher than that induced by H_2_O_2_ (Fig. 4h). Accordingly, AR may play a protective role in RA condition, it is responsible for the reduction of toxic aldehydes, and AR inhibition-induced accumulation of 4-HNE might be the major reason for exaggerating the activation of autoimmune and inflammatory responses.

### Hind joint AAV-mediated overexpression of AR does not attenuate the arthritic condition in AIA rats

**Figure 5:**
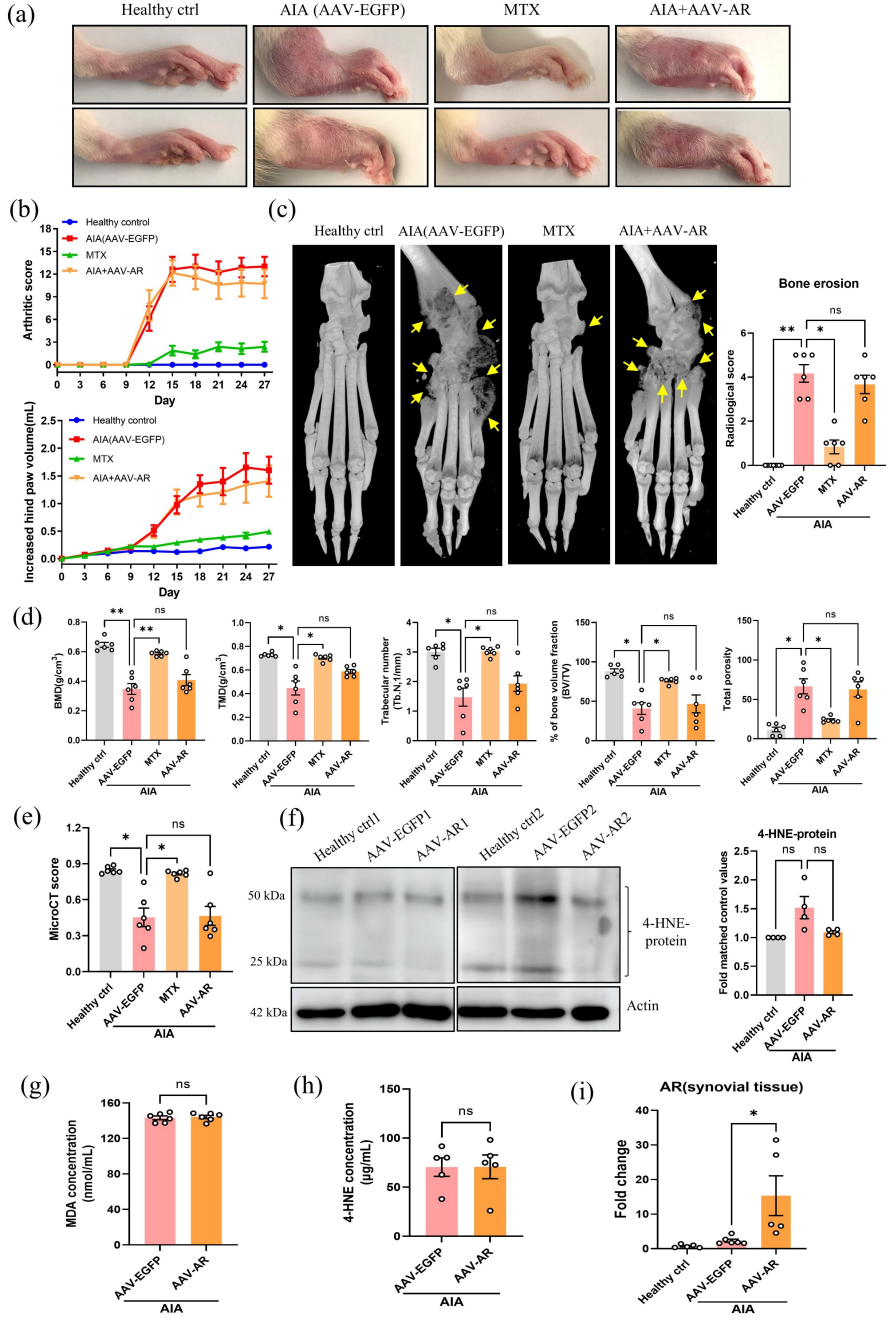
** Hind joint AAV-mediated overexpression of AR does not attenuate the arthritic condition in AIA rats. (a)** Representative images of swollen hind paw of AAV-AR-injected AIA rats. Hind paw swelling images were captured on Day 27.** (b)** The hind paw swelling and arthritis scores of AAV-AR injected AIA rats.** (c)** Representative micro-CT radiographic images and radiological scores of damaged hind joint bone of AIA rats with AAV-mediated overexpression of AR. Yellow arrows indicate the region of severe bone erosion. **(d)** Five disease-related indexes of micro-CT analyses of the BMD, BV/TV, TMD, Tb. N, and the percentage of total porosity of AAV-AR-injected AIA rats. **(e)** The micro-CT scores of AIA rats with AAV-mediated overexpression of AR. Micro-CT scores were obtained from five disease-related indexes.** (f)** Expression level of 4-HNE-protein conjugate in AIA rats with AAV-mediated overexpression of AR. Synovial tissues were harvested from healthy, AIA model and AIA with AAV-mediated AR overexpression groups for extraction of protein and Western blotting using antibodies against 4-HNE-protein conjugate and β-actin (loading control). Histogram show the quantitation of 4-HNE-protein conjugate expression relative to the healthy controls.** (g)** The changes of the serum concentration of MDA in AIA rats injected with AAV-AR.** (h)** The changes of the serum concentration of 4-HNE in AIA rats injected with AAV-AR. **(i)** Gene expression level of AR in rat synovium from healthy, AIA model and AAV-AR overexpression group. The data are expressed as the means ± SEM (n ≥3). *P<0.05, **P<0.01, ***P<0.001 significant different compared with AIA models.

Since AR may exhibit a protective role during the pathogenesis of RA, we further addressed whether adeno-associated virus (AAV)-mediated overexpression of AR would demonstrate an anti-arthritic effect in AIA model. Severe erythema, joint rigidity and swelling were observed in the hind paws of AAV-EGFP injected AIA rats (vehicle control) and AAV-AR (Fig. 5a). There was no significant difference in arthritic scores and hind paw volume between AIA rats injected with AAV-EGFP or AAV-AR (Fig. 5b). Analysis of micro-CT data further demonstrated that overexpression of AR in hind joint did not change the conditions of bone destruction and cartilage loss (Fig. 5c-e). Besides, no significant changes in gene expression of inflammatory makers were found in PBMCs of AIA rats injected with AAV-AR when compared with vehicle control (Fig. S1b). Importantly, AAV-AR injection only slightly reduced the content of 4-HNE protein conjugate in synovial tissue (Fig. 5f, with no statistical significance), though 4-HNE is an abundant substrate for AR. ELISA assay further confirmed the comparable serum concentrations of 4-HNE and MDA between AIA model and AIA rats injected with AAV-AR (Fig. 5g and 5h). With qPCR confirmation, we verified the AAV-mediated overexpression of AR in joint tissues of AIA rats (Fig. 5i). Of note, the upregulation of AR in AIA rats (Fig. 1c and 5i) was possibly responsible for detoxifying excessive saturated and unsaturated aldehydes, and thus only a minimal increase in 4-HNE content was observed in AIA rats (Fig. 4c-f and Fig. 5f). Collectively, overexpression of AR in joints of AIA rats did not exaggerate or attenuate the arthritic condition, supporting the view that high expression of AR may not be a dominant contributor in AIA condition, but is an important part of antioxidative defense mechanism, preventing the harmful aldehydes, such as 4-HNE, from excessive accumulation during chronic inflammation and oxidative stress.

### Anti-oxidant NAC combined with AR inhibitor, epalrestat markedly suppresses arthritic condition in AIA rats

Inconsistent results were found in the effect of epalrestat on inflammatory response *in vitro* and *in vivo*, implying that the outcomes of AR inhibition may be highly context dependent. It is imperative to find a way to eliminate the side effect of epalrestat on the development of RA, considering the patients with RA may also suffer from diabetic complications, and may need epalrestat for the treatment. Given the close relation between RA and oxidative stress 58, the increased level of 4-HNE induced by epalrestat treatment, and obviously decreased content of 4-HNE-protein by NAC 59, 60, it is worth to investigate the anti-arthritic effect of combined therapy of epalrestat with NAC.

**Figure 6:**
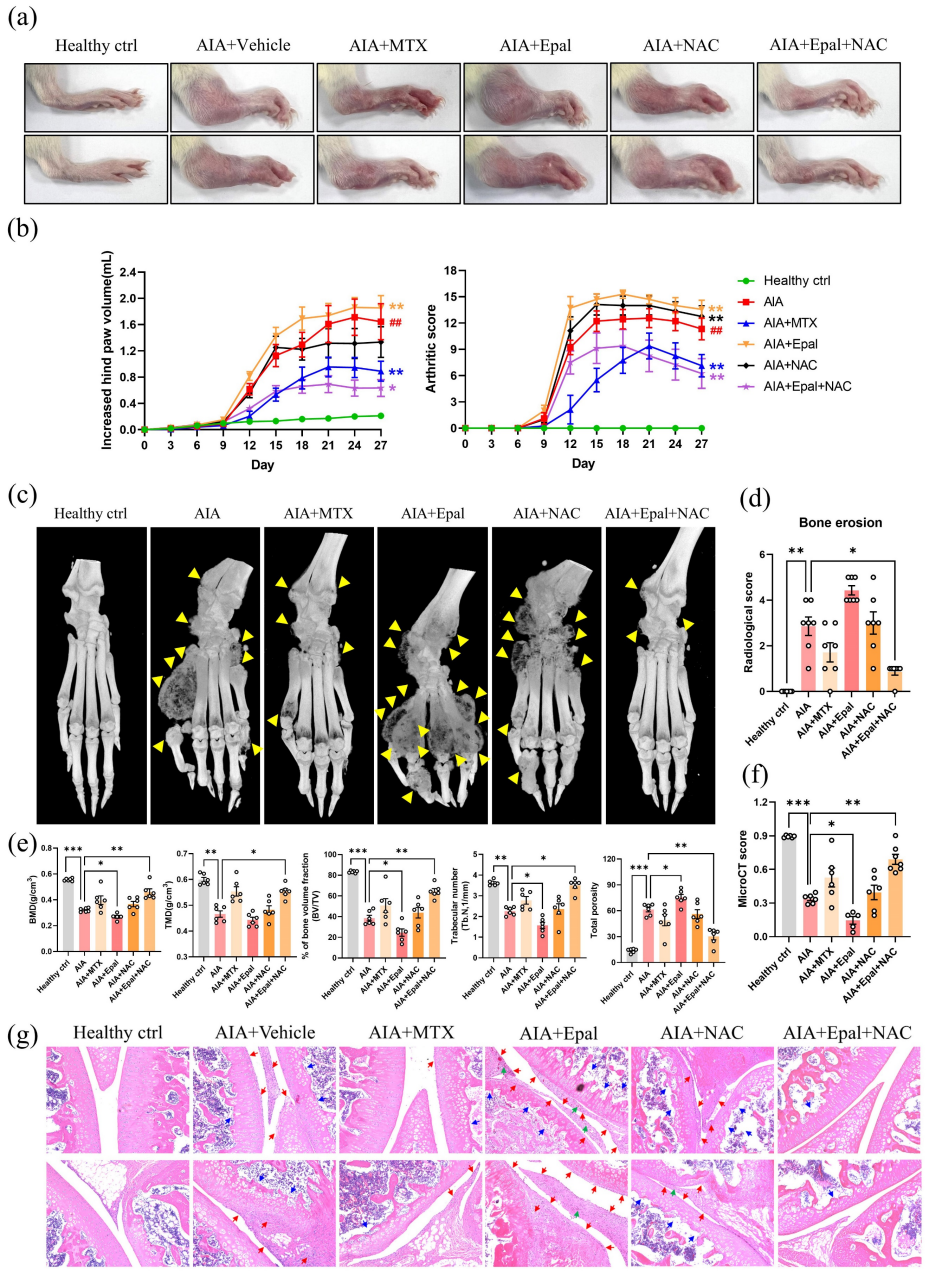
** Anti-oxidant, N-acetyl-L-cysteine (NAC) combined with AR inhibitor markedly suppresses arthritic condition in AIA rats. (a)** Representative images of swollen hind paw of AIA rats with Epal/NAC combined treatment. Hind paw swelling images were captured on Day 27. **(b)** The arthritic scores and hind paw swelling of AIA rats with Epal/NAC combined treatment. **(c)** Representative micro-CT radiographic images of AIA rats with Epal/NAC combined treatment. Yellow arrows indicate the region of severe bone erosion. **(d)** Radiological scores of damaged hind joint bone of AIA rats with Epal/NAC combined treatment. **(e)** Five disease-related indexes of micro-CT analyses of the BMD, BV/TV, TMD, Tb. N, and the percentage of total porosity of AIA rats with Epal/NAC combined treatment. **(f)** The micro-CT scores of AIA rats with Epal/NAC combined treatment. **(g)** H & E staining of histological sections of knee joint tissues from AIA rats with Epal/NAC combined treatment. Blue arrows indicate bone loss; red arrows indicate hyperplasia of synovial membrane; green arrows indicate the pannus formation (Magnification, ×100).

As shown in Fig. 6a&b, NAC alone had no obvious effect on the arthritic condition in AIA rats, the arthritic score of NAC treatment group was comparable to that of vehicle treated AIA rats. Epalrestat alone exaggerated the swelling, erythema and bone destruction in AIA rats (Fig. 6a-f) consistent to the aforementioned results (Fig. 2a-f). However, the severe arthritic conditions induced by epalrestat were significantly reversed by the co-treatment of NAC (Fig. 6a-f). Apparently, micro-CT analysis of hind paws illustrated the obvious therapeutic effects of epalrestat combined with NAC in AIA rats (Fig. 6c-f). Moreover, histopathological analysis showed that arthritic characteristics such as hyperplasia of synoviocytes with infiltration of neutrophils, proliferation of interstitial fibroblast accompanied with neovascularization, loss of cartilage in joint tissues were barely observed in AIA rats with combined treatment of epalrestat/NAC (Fig. 6g).

**Figure 7:**
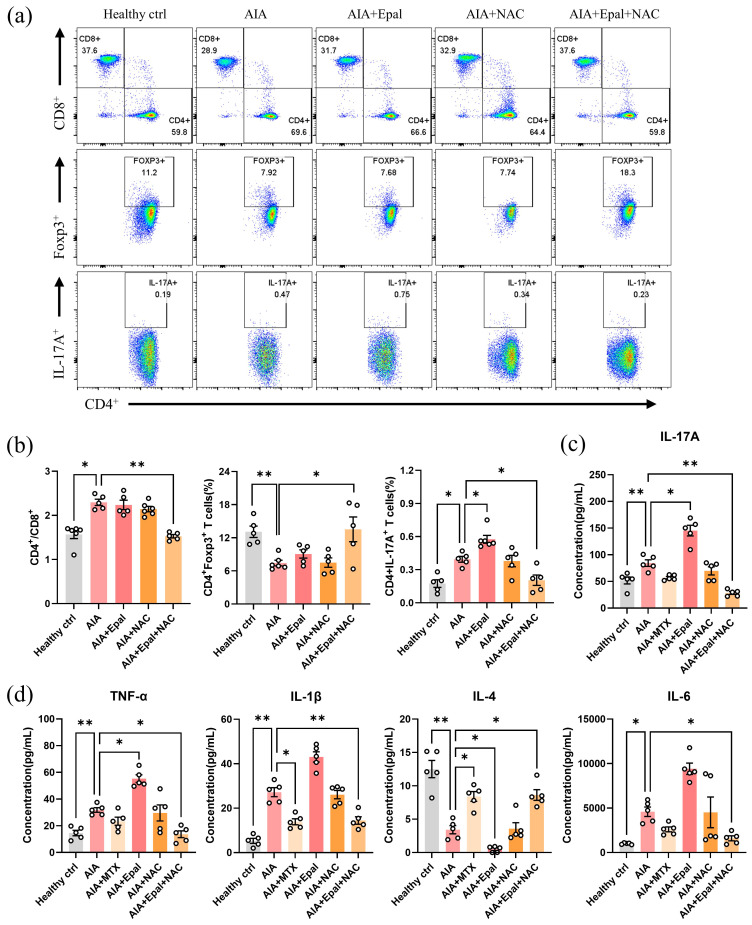
** NAC reverses the epalrestat-induced autoimmune response in AIA rats.** Blood lymphocytes were harvested from epalrestat and NAC combined treated AIA rats for flow cytometry analysis using fluorescent antibodies against CD45, CD3, CD4, CD8, IL-17A and Foxp3.** (a)** Representative flow cytometry images show the percentage of CD4^+^ and CD8^+^ T lymphocytes gated on CD3^+^ T lymphocytes, and the percentage of the Foxp3^+^ T cell or IL-17A^+^ T cells gated on CD4^+^ T lymphocytes.** (b)** The quantitative bar charts show the ratio of CD4^+^ / CD8^+^ T cells, and the percentage of IL-17A^+^ T cells or Foxp3^+^ T cells among CD4^+^ T cells.** (c&d)** Co-treatment of NAC reversed the changed serum concentration of cytokines in AIA rats. The concentration of different cytokines was measured using LEGENDplex^TM^ Customer Panel. The data are presented as the means ± SEM (n ≥5). *P<0.05, **P<0.01, ***P<0.001, compared with healthy control or vehicle control.

Evaluation of immune cells in rat peripheral blood lymphocytes showed comparable ratio of CD4^+^/CD8^+^, and the frequency of Foxp3^+^ and IL-17A^+^ in CD4^+^ T cells between NAC- and vehicle-treated AIA group (Fig. 7a and 7b), suggesting that NAC treatment alone did not alleviate the autoimmune response of animals. Interestingly, the combination of epalrestat and NAC significantly increased the percentage of Foxp3^+^ Treg cells, decreased the ratio of CD4^+^/CD8^+^ and the percentage of IL-17A^+^ Th17 cells when compared with AIA models (Fig. 7a and b). Notably, epalrestat-induced increase in Th17 cells was markedly reversed by the co-treatment of NAC. Consistent with the above results, lower concentrations of IL-1β, IL-6, IL-17A and TNF-α, were detected in the serum of combined epalrestat-NAC treatment group (Fig. 7c and 7d). Compared to AIA model, the concentration of anti-inflammatory cytokines IL-4 was significantly decreased in epalrestat treated group, but was markedly increased in combined treatment group, comparable to MTX treatment group. Collectively, these findings demonstrated that the supplement of anti-oxidant may override the side effect of AR inhibition by epalrestat to exhibit excellent anti-arthritic and anti-inflammatory effect, regardless of the anti-oxidative role of AR in RA development.

### NAC reversed the excessive accumulation of 4-HNE induced by epalrestat

**Figure 8:**
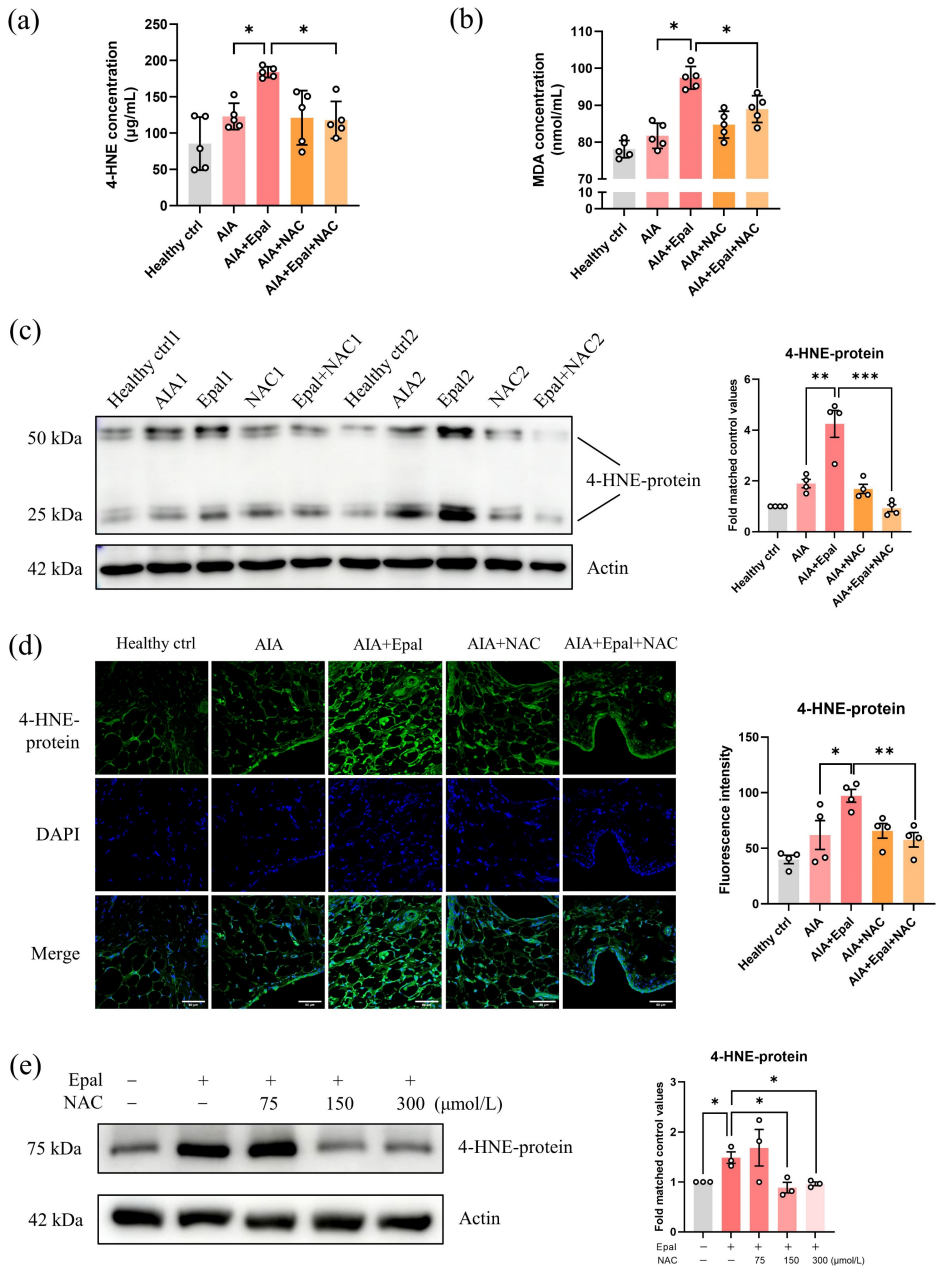
** NAC decreases the excessive accumulation of 4-HNE induced by epalrestat. (a)** The changes of the serum concentration of 4-HNE in the AIA rats with combined treatment of epalrestat and NAC. **(b)** The changes of the serum concentration of MDA in AIA rats with combined treatment of epalrestat and NAC. **(c)** Expression level of 4-HNE-protein conjugate in epalrestat and NAC combined treated AIA rats, bar chart shows the quantitation of 4-HNE-protein conjugate expression relative to the healthy controls. **(d)** Immunofluorescence staining of 4-HNE-protein conjugate in the knee synovium of AIA rats with epalrestat and NAC combined treatment, scale bar: 60 μm, bar chart shows the *Image J* quantitation of the fluorescence intensity of 4-HNE-protein conjugate in the synovium tissues. **(e)** Expression level of 4-HNE-protein conjugate in epalrestat and NAC combined treated RAFLS. Cells were treated with 50 μmol/L Epalrestat overnight with or without 75-300 μmol/L NAC. The data are presented as the means ± SEM (n ≥ 3). *P<0.05, **P<0.01, significant different when compared with vehicle-treated AIA group.

Glutathione (GSH) is an important antioxidative participator responsible for the metabolism of toxic aldehydes including 4-HNE 61. As a precursor of reduced GSH, NAC has been reported to downregulate the content of 4-HNE and MDA 59, 60. Consistently, co-treatment with NAC dramatically reduced the high serum concentrations of 4-HNE and MDA induced by epalrestat as expected (Fig. 8a and 8b). Immunofluorescent staining and Western blot further confirmed that epalrestat alone caused significant accumulation of 4-HNE conjugated protein in synovial tissues of AIA rats, and the increase content of 4-HNE was significantly reversed by NAC co-treatment with a comparable expression level to healthy controls (Fig. 8c and 8d). Consistent with the *in vivo* study, co-treatment of NAC *in vitro* obviously downregulated epalrestat induced overexpression of 4-HNE-protein conjugate in RAFLS (Fig. 8e). Altogether, these results clarified that the excessive accumulation of 4-HNE caused by AR inhibition by epalrestat could be eliminated by NAC, therefore illustrating an anti-arthritic effect in AIA rats as depicted in Fig. 6a-f.

## Discussion

In the past decades, ubiquitously expressed enzyme, AR, which converts excess glucose to sorbitol, has been considered to be implicated in the etiology of secondary complications of diabetes such as diabetic neuropathy (DN), thus the inhibition of AR by epalrestat was used against DN clinically. On the other hand, it is well known that AR has multiple substrates including 4-HNE, and is responsible for reducing unsaturated toxic aldehydes generated from lipid peroxidation to inactive alcohol 62. In the present study, our experimental data supported this view that the enzymatic activity of AR is essential for removing toxic aldehydes and reducing oxidative stress in RA, an autoimmune disease closely associated with chronic inflammation and oxidative damage. Similarly, this detoxified nature of AR was also reported in giant cell arteritis, a systemic vasculitis 35. The increased expression of AR catalyzes the excessive aldehydes including 4-HNE, to maintain homeostasis and prevent the harmful effect of these highly reactive aldehydes in RA condition. However, AAV-mediated overexpression of AR could not ameliorate the arthritic condition of AIA rats, probably due to existing activated and inactivated forms of AR 63. Thus, exogenous supply of AR may not further convert to the activated state for aldehyde detoxification.

Highly reactive aldehydes especially 4-HNE contribute to chronic inflammation *via* mediating numbers of signaling pathways through modification of proteins, DNA and phospholipids 64. Since antibodies against 4-HNE and MDA have been detected in many autoimmune diseases including RA 65, 66, suggesting that these aldehydes can act as auto-antigens, together with its auto-antibodies are certainly robust activators of the immune system and engaged in the development or at least exacerbation of RA. Consistently, MDA- and 4-HNE-derived antigens exaggerates disease severity *via* CD4^+^ T cell recruitment and Th1 cell activation in hepatic inflammation 67. 4-HNE-modified antigen presenting to B cells and T cells causes rapid onset of autoimmune reactions 68. We demonstrated that epalrestat treatment aggravated the disease severity of RA with exaggerated immune responses, possibly resulting from the excessive accumulation of 4-HNE. Our findings confirmed the detrimental roles of 4-HNE and other possible aldehydes in RA, which was consistent with Biniecka's study 69. Therefore, epalrestat should be prescribed with great attention for the diabetic patients with RA or other autoimmune diseases, considering the excessive accumulation of 4-HNE due to AR inhibition by epalrestat. For those patients, it is necessary to take a safety precaution when using epalrestat for treatment of diabetic complications. Thus, we verified that the increase of 4-HNE arising from epalrestat-induced AR inhibition can be eliminated by NAC, and the combined treatment exhibited great anti-arthritic effect in AIA model. A recent study demonstrated that alpha lipoic acid (ALA), an antioxidant, combined with epalrestat is more effective with reduced inflammatory and oxidative markers than epalrestat monotherapy in treating DN clinically 70. Obviously, this combined strategy implicitly coincided with our view that the anti-inflammatory property of epalrestat may be diminished or overridden by the side effects of excessive accumulation of 4-HNE due to AR inhibition, and co-treatment with antioxidant helps removal of aldehydes, and potentiates the anti-inflammatory effect of epalrestat.

Emerging evidence showed that inhibition of AR suppresses inflammatory responses in multiple diseases 71. Interestingly, despite the fact that epalrestat augmented the immune response and inflammation in AIA rats, the transcriptional level of TNF-α and IL-1β were significantly down-regulated in the PBMCs of epalrestat-treated AIA rats when compared with AIA models (Fig. S1c). These opposite results may be explained by the fact that the transcription is an acute response. AIA model is a RA model with the activation of T cells and neutrophils which does not last more than a month 72. During the later exposure to CFA, the robust immune response tended to be subsided as evidenced by the gradually decreased arthritic score after day 21 (Fig. 2b), the anti-inflammatory property of epalrestat was dominant, and thus suppressed the transcription of inflammatory cytokines, which was consistent with our *in vitro* data (Fig. 1g). Recently, juglanin, a natural product with partly AR inhibitory 73 and antioxidant activities 74, has been reported to alleviate arthritic phenotype in AIA rats through inactivation of NF-κB and decrease of oxidative stress 75. Surprisingly, epalrestat with the specific ARI activity leads to more severe arthritic symptoms but co-treatment with NAC, exhibits anti-arthritic and -inflammatory effects in AIA rats. This suggests that Juglanin may act more as anti-oxidant as evidenced by the down-regulated concentration of MDA and the upregulated level of GSH after its administration. Therefore, combined therapy of epalrestat and antioxidant NAC may be considered as a new or adjuvant strategy in treating RA.

Conventional nonsteroidal anti-inflammatory drugs (NSAIDs) and glucocorticoids are mainstays in controlling RA-related inflammation and severe pain. However, long-term use of NSAIDs may result in gastrointestinal abnormalities, which has been accounted for the considerable rate of death among RA patients 76. Equally, high dose usages of glucocorticoids are associated with osteoporosis or cardiovascular risk 77. Conversely, the minor adverse effects induced by epalrestat, such as nausea, vomiting, and diarrhea, can generally be resolved spontaneously by modifying the dosage, and thus epalrestat appeared to be well-tolerated for long-term treatment 78-80. These evidences suggested that epalrestat is highly suitable for controlling chronic inflammation associated with RA. Besides, RA is always related to persistent pain, which severely decreases the quality of life in RA patients. It is possible that RA patients may have peripheral neuropathic pain condition 81, 82, in which epalrestat has been used to alleviate neuropathic pain in diabetic patients 83. Therefore, epalrestat combined with NAC may have potentiating effect in alleviating neuropathic pain associated with RA.

Patients with RA have a significantly increased risk of cardiovascular diseases, and substantial epidemiological data revealed that about 50% of mortality is attributable to cardiovascular disease, especially atherosclerosis in RA patients 84, 85. RA patients usually with high levels of low-density lipoproteins and cholesterol that along with oxidative stress lead to lipid peroxidation, which further contributes to the development of RA and the susceptibility of atherosclerosis 86, 87. Besides, medicines like cyclooxygenase inhibitors and NSAID may detrimentally affect lipid metabolism and increase adverse cardiovascular events 88. Although the etiology of atherosclerosis is complicated, growing evidence indicated that the increased lipid peroxidative productions, especially 4-HNE, are involved in endothelial dysfunction and pathogenesis of atherosclerosis 61, 89, 90. Our experimental data demonstrated the increased contents of 4-HNE and MDA in AIA model, and similar trends were commonly observed in RA patients 40. High levels of 4-HNE and other lipid peroxidative products along with chronic inflammation under RA-related oxidative conditions are definitely high-risk factors of atherosclerosis. Therefore, monitoring the concentration of 4-HNE or other lipid peroxidative products in blood or urine would be an important indicator for atherosclerosis risk management in RA patients.

Lipid peroxidation and its end-products are commonly considered as biomarkers and potential therapeutic targets in atherosclerosis. The rate of lipid peroxidation products metabolism is critically dependent on GSH level and relevant enzymes, reflecting the antioxidant capacity of different pathological states. Reduced GSH is easily reacted with 4-HNE to form less toxic GSH-conjugate of 4-HNE (GS-HNE) 91, 92. Similar to aforementioned AR substrate 4-HNE, its conjugate GS-HNE can also be converted by AR to glutathionyl-1,4 dihydroxy nonene (GS-DHN), which has been verified to be a great activator of inflammatory pathway including NF-κB, MAPK, PI3K and AP-1 20, 21. Inhibition of AR by epalrestat may suppress systemic inflammation *via* reducing the production of GS-DHN, but lead to the buildup of toxic aldehydes, which may contribute to autoimmune and inflammatory conditions in AIA rats. On the other hand, co-treatment with epalrestat with NAC, can also increase GSH level, and reduce 4-HNE accumulation, leading to the combined therapeutic effect *via* AR inhibition and anti-oxidation in RA. Given the anti-inflammatory effect and the reduction of 4-HNE and MDA levels, epalrestat-NAC combined therapy may protect against the development of atherosclerosis in RA patients. Moreover, upregulation of AR in human macrophage has been proved to facilitate inflammation and associate with atherosclerosis 93. Inhibition of AR diminishes mitogen-induced DNA synthesis and cell proliferation 94, 95, since AR is known to increase the proliferation of vascular smooth muscle cells (VSMCs), which is an important factor involved in the pathogenesis of atherosclerosis 36. These studies also confirmed the beneficial effects of AR inhibition in not only RA but also in the management of atherosclerosis.

## Conclusion

Taken together, the overexpression of AR in RA may act to reduce aldehydes, such as 4-HNE, and act as an antioxidant. The reduced products like GS-DHN generated from AR catalysis and the excessive accumulation of aldehydes resulted from AR inhibition are both great inducers of inflammation, which make the results of AR inhibition by epalrestat highly context-dependent. Epalrestat monotherapy leads to more significant buildup of 4-HNE, and worse articular joint morphology in AIA rats. Interestingly, epalrestat co-treatment with NAC exhibits anti-arthritic effect by inhibiting inflammatory and immune responses and reducing toxic aldehydes, suggesting a potential way in treating RA. For patients with RA and DN, this therapeutic strategy could be considered as a potential and safe approach, leading to a simultaneous improvement of both inflammation and glucometabolic. Obviously, further clinical studies are required to verified the efficacy of this combined strategy in patients with both RA and diabetic complications in coming future.

## Methods and materials

### Rheumatoid arthritis patients and samples

All subjects were provided written, informed consent, and all the protocols were approved by Ethics Committee of Macau Medical Science & Technology Research Association (NO. 2021OCT01). RA patients (n=26) and healthy donors (n=14) were recruited from Centro Hospitalar Conde de São Januário (Macau, China) between October 2021 and October 2022. Volunteers with diabetes were excluded in current study. All of the epidemiological investigation and classification of the individuals were carried out according to the principle of European League Against Rheumatism (EULAR). Clinical characteristics of RA patients (rheumatoid factor (RF)-positive RA), patients categorized by remission (DAS28<2.6), low disease activity (2.6 ⩽ DAS28 ⩽ 3.2), moderate disease activity (3.2 < DAS28 ⩽ 5.1), high disease activity (DAS28 > 5.1) criteria included 11 patients, 5 patients, 2 patients and 1 patient respectively.) and healthy donors are summarized in Table 1.

### Real-time quantitative PCR

Total RNA from PBMCs or cells was extracted using FavorPrep™ Total RNA purification mini kit (Favorgen Biotech, Taiwan) following the manufacturer's instructions. Total RNA from synovial tissue was extracted with TRIzol (Invitrogen, USA). Synovial tissues were frozen and grind in liquid nitrogen, then 1 ml of TRIzol was added and mixed sufficiently, 100 μl of chloroform was used as extracting solvent, the mixture was centrifuged at 4 °C and 12000 g for 15 min. Collected the supernatant and precipitated it with isopropanol; this mixture was then centrifuged at 12000 g for 15 min. The white precipitate was then washed with 70-80% cold ethanol before dissolution with 40 µl of RNase-free water. The quality and concentration of RNA was determined using NanoDrop 2000c Spectrophotometer (Thermo, USA). The synthesis of cDNA is followed the instruction of SuperScript® VILO™ Master Mix kit (Invitrogen, USA). Real-time quantitative PCR was carried out on ViiA™ 7 real-time PCR system (Applied Biosytems, USA). PCR reaction mixture (total 10 µl) contained 0.5 µl of template cDNA, 0.2 µl each of the forward and reverse primers, 5 µl of FastStart Universal SYBR-Green Master Rox (Roche Diagnostics, Indianapolis, IN, USA), 0.2 µl Passive Reference Dye (Roche Diagnostics, USA) and 3.9 µl of H_2_O. The procedure of real-time RCR is 50˚C for 2 min, 95˚C for 10 min, followed by 40 cycles of 95˚C for 30 sec, 60˚C for 60 sec. Expression level of mRNA was normalized with β-actin and calculated using the 2^-ΔΔCT^ method. Ranges of values will be obtained in at least three parallel analyses with <5% of the means. Primer sequences are designed as below:

### Cytotoxicity assays

The cytotoxicity of Epalrestat towards our cellular models was assessed with the use of 3-(4,5-dimethylthiazol-2-yl)-2,5-diphenyltetrazolium bromide (MTT) (5 mg/ml) assay. Briefly, RAFLS (6000 cells/well) were cultured in 96-well plates before treated by epalrestat. Cells were then treated with different concentrations of epalrestat (12.5-200 μmol/L) for 24 h. 10 μL of MTT was added and incubated at 37℃ for 4 hrs followed by the addition of 100 μL solubilization buffer (10% SDS in 0.01 mol/L HCl) and incubated at 37℃ overnight. The OD value was measured at A570 nm. The proportion of cell viability was calculated as follow: Cell viability (%) = A_treated_/_Acontrol_×100.

### Induction and assessment of AIA model

All animal care and experimental protocols were approved by the Animal Ethical Committee of Department of Health and Supervision, Macau Special Administrative Region of China, and carried out accroding to the “Institutional Animal Care and User Committee guidelines” of the Macau University of Science and Technology. For the induction of AIA model, male Sprague Dawley rats (weighing 120±20 g) were obtained from ZHUHAI BESTEST BIO-TECH CO., LTD. Animals were raised in a humidity- and temperature- controlled environment under a 12 h day/night cycle. Arthritis was induced by inoculation with complete Freund's adjuvant (CFA) in rats. Non-viable desiccated *Mycobacterium tuberculosis* (BD, USA) was emulsified in mineral oil (Sigma, USA). AIA rats were induced by intradermal injected 0.1 ml of the 5 mg/ml emulsion at the base of the tail on day 0. Rats intradermal injected with 0.1 ml saline solution at the base of tail were used as healthy control.

To investigate the effect of epalrestat on AIA rats, the CFA-induced animals were randomly assigned into three groups (n=5-7). From day 0, vehicle, MTX (7.6 mg/kg, once a week) or Epal (15.5 mg/kg, once a day) were oral administrated. As a clinically approved drug, the normal dosage of Epalrestat for adults is 150 mg per day, and in current study, to explore the influence of this dosage of Epalrestat on the development of RA, we specifically use a dose of 15.5 mg/kg according to the principle of dose conversion between animal and human 96.

To explore the potential role of AR in RA, adeno-associated virus serotype 2/5 packaging CMV/rAkr1b1/P2A/EGFP vector (AAV-AR) or CMV/EGFP vector (AAV-EGFP) were generated by Cyagen, Inc. (USA). SD rats were divided into 4 groups (n=5-7) as follows: (1) healthy control, (2) AIA model (AAV-EGFP), (3) AIA + AAV-AR, (4) AIA + MTX (7.6 mg/kg/week). AAV-EGFP (2.5×10^10^ PFU) or AAV-AR (2.5×10^10^ PFU) were intra-articular injected 7 days before AIA induction.

To identify the effect of Epal combined with NAC on AIA rats, the CFA-induced animals were randomly divided into 5 groups (n=5-7). From the day 0, vehical, MTX (7.6 mg/kg/week), Epal (15.5 mg/kg/day), NAC (100 mg/kg/day), or Epal (15.5 mg/kg/day) plus NAC (100 mg/kg/day) were administered. MTX and Epal was oral administrated, NAC was intraperitoneal injection.

Joint swelling was observed every day, the arthritis scores and the increased hind paw volume were evaluated and measured every 3 days from AIA induction (Day 0) until the animals were euthanasia. Joint swelling was measured using plethysmometer (Ugo Basile, Italy), the increased paw volume of each rat was calculated by the average of increased paw volume of left and right hind paw. Arthritic scores for each paw of the rats were evaluated individually on a scale from 0 to 4 according to the arthritis index described in our previously published paper 53.

### Micro CT analysis

At the end of animal experiment, rats were euthanasia. The left hind paws were amputated and fixed in 4% PFA, then scanned it by microCT scanner (SkyScan 1176, Bruker, Belgium) using the following parameters: 35 μm resolution, 85 kV, 385 μA, 65 ms exposure time, 0.7° rotation step in 360°, and a 1 mm Al filter. The micro-CT images were reconstructed by NRecon software (Bruker-microCT, Belgium). The microstructure of hind paw and diseases-related index was analyzed at the same area using CTvox software. MicroCT score was calculated using the following formula: (Acquired value - minimum value) / (maximum value - minimum value), according to five disease-related index of microCT analysis for calcaneus (bone mineral density, bone volume fraction, cortical mineral density, trabecular number, and total porosity). The final microCT scores is calculated by averaging these five disease-related indicators. (Micro-CT score: 0-0.2, mutilating; 0.2-0.4, severe; 0.4-0.6, moderate; 0.6-0.8, mild; 0.8-1, normal.).

The severity of bone erosion was evaluated according to the radiological scores system described in Table 3.

### Immunofluorescent staining and hematoxylin and eosin (H&E) staining

Knee joint tissue from all groups were fixed in 4% paraformaldehyde for at least 24 h, and subjected to dehydrated and embedded in paraffin at 60 ℃. 5 μm thick sections were dehydrated, deparaffinized, rehydrated. Sections of rat synovial tissue were double incubated with anti-AR (1:250, Santa Cruz) and anti-Vimentin (1:250, Invitrogen) or anti-4-HNE-protein conjugate (1:250, Invitrogen) primary antibodies at 4 ℃ overnight. Then incubated with fluorescent secondary antibody (anti-rabbit Alexa 555 or anti-mouse FITC, Beyotime) in the dark at room temperature for 2 h. Finally, mounted with FluorSave^TM^ Reagent (Millipore). Fluorescent images were captured with TCS SP8 DLS confocal microscope (Leica, German). For histological analysis, rat joint sections from all groups were stained with hematoxylin and eosin, and the pictures of stained joint tissues were captured by LED optical microscope (Leica DM2500).

### Flow cytometry

Blood was collected from AIA rats; peripheral blood mononuclear cells (PBMCs) were obtained by using Ficoll density gradient centrifugation kit (GE Healthcare). Cells were washed twice with phosphate buffered saline. For the assessment of Th17 cells, PBMCs were stimulated by Cell Activation Cocktail with brefeldin A (BioLegend, USA) for 6 hours. Then cells were stained with APC/Cy7 conjugated anti-CD45, FITC conjugated anti-CD3, PerCP/Cy5 conjugated anti-CD4, APC conjugated anti-CD8 antibody for 1 hour in the dark at 4 ℃. Subsequently, cells were fixed and permeabilized using Cyto-Fast^TM^ Fix/Perm Buffer Set (BioLegend, USA) for 30 min in the dark at 4 ℃. Then cells were washed and labelled intracellularly with PE/cy7 conjugated anti-IL-17A antibody for 1 hour in the dark at 4 ℃. Similarly, for the assessment of Treg cells, PBMCs were stained with APC/Cy7 conjugated anti-CD45, FITC conjugated anti-CD3, PerCP/Cy5 conjugated anti-CD4, APC conjugated anti-CD8 antibody for 1 hour in the dark at 4 ℃. After washed, fixed and permeabilized using True-Nuclear^TM^ Transcription Factor Buffer Set (BioLegend, USA), cells were washed before staining with PE-A conjugated anti-Foxp3 antibody intracellularly. Cells were then washed and resuspended in 200 cL flow cytometry staining buffer and analyzed using FACSAria III cell sorter (BD Biosciences, USA). Gating strategies used for detection of Th17 cells and Treg cells are demonstrated in Fig. S1d). The cytometric data were analyzed with FlowJo v10 software (TreeStar, Inc.). Except for PE/cy7 conjugated anti-IL-17A antibody purchased from Invitrogen (Thermo, USA), other antibodies were obtained from Biolegend.

### Western blotting

Synovial tissues or RAFLSs were lysed with RIPA lysis buffer (1× Protease inhibitor cocktail from Roche) for 15-30 min. Supernatant were collected after centrifuged at 4 ℃, 12000 rpm for 15 min. The concentration of protein was determined by spectrophotometer at a wavelength of 595 nm. 15 μg of protein was loaded and separated by 8-10% SDS-PAGE, then transferred to PVDF membranes (Bio-Rad, USA) and blocked using 5% non-fat milk for 1 hour at room temperature. The membranes were incubated with anti-4-HNE protein (1:1000; Invitrogen) conjugate, anti-p-p65 (1:1000; CST), anti-p65(1:1000; CST) and anti-β-actin (1:1000; Santa Cruz Biotechnology) or GAPDH (1:1000; Santa Cruz Biotechnology) antibody at 4 ℃ overnight. After washed with TBST for three times, the membranes were incubated with anti-mouse secondary antibodies (1:3000; Cell signaling techonlogy) or anti-rabbit secondary antibodies (1:3000; Cell signaling technology) for 2 hours at room temperature. SuperSignal West Femto Maximum Sensitivity Substrate Kit (Thermo) was used to detect the bands with Amersham Imager 800 (GE) Imaging System. Quantitative analysis was performed using *ImageJ* software.

### ELISA

#### Detection of the concentration of cytokines

The concentration of anti-inflammatory and inflammatory cytokines was detected using LEGENDplex^TM^ Rat Inflammatory Panel (Biolegend) or LEGENDplex^TM^ Customer Panel (Biolegend) following the manufacturer's protocol and acquired *via* BD FACSAria III cell sorter (BD Biosciences, USA).

#### Detection of the concentration of MDA and 4-HNE

The concentration of MDA and 4-HNE was detected using lipid peroxidation (MDA) Assay kit (abcam, USA) and lipid peroxidation (4-HNE) Assay kit (abcam, USA) according to the manufacturer's instructions. The OD value or fluorescence signal was determined by SpectraMax Paradigm Multi-Mode Microplate Reader (MOLECULAR DEVICES, USA).

### Statistical analysis

Statistical analysis was performed using GraphPad Prism 9 software. The results were expressed as mean ± SEM as indicated. The difference was considered statistically significant when the *p* values were less than 0.05. One-way ANOVA analysis or student's t-test were used for the comparison among different groups.

## Supplementary Material

Supplementary figure.

## Figures and Tables

**Figure 9:**
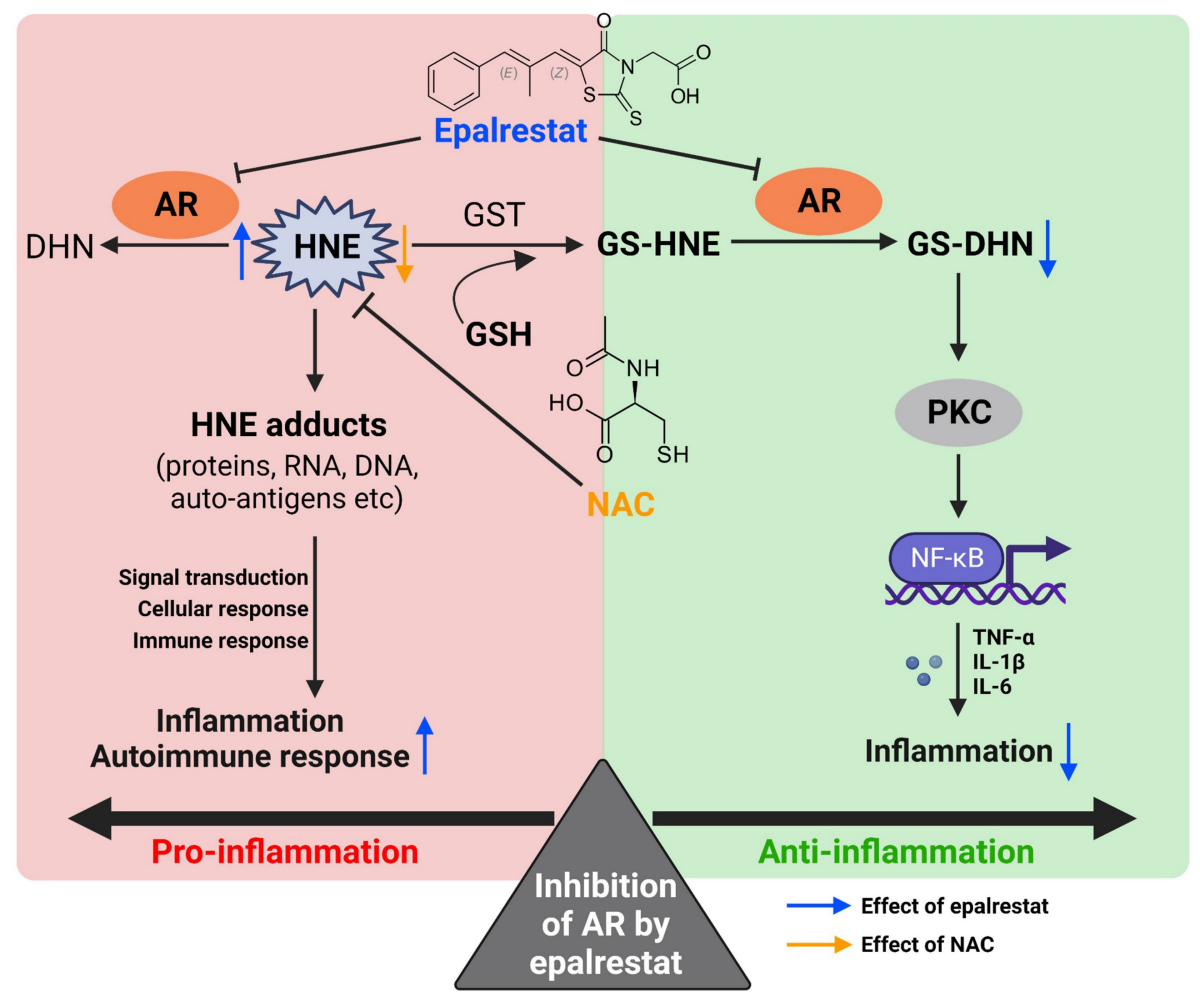
** Bifacial effect of AR inhibition by epalrestat.** Inhibition of AR can reduce the generation of GS-DNH, and thus exhibit anti-inflammatory effect (green part). On the other hand, inhibition of AR by epalrestat increases the content of free HNE and its adducts, which accelerates autoimmune and inflammatory responses (red part). Co-treatment with NAC suppresses the buildup of 4-HNE induced by epalrestat-mediated AR inhibition, thus exhibiting combined anti-arthritic effect.

**Table 1:** The baseline data of RA patients and healthy volunteers

	RA (N=26)	Healthy (N=14)
**Age (years (mean (range)))**	62(47-81)	56(36-71)
**Sex (N (F/M))**	23/3	9/5
**ESR (mm/h (mean (range)))**	16.4(3.6-31.2)	NA
**CRP (mg/L (mean (range)))**	11.5(3.3-39.5)	NA
**DAS28 score (mean (range))**	2.7(1.8-5.2)	NA
**RF (IU/mL (mean (range)))**	236.3(106.4-399.2)	NA

CRP, C-reactive protein; ESR, Erythrocyte sedimentation rate; RF, Rheumatoid factors; NA, not assessed.

**Table 2:** Sequence of primer

Primer	Forward	Reverse
Human-AR	GGGTTGGGTACCTGGAAGTC	TGGTACACATGGGCACAGTC
Human-TNF-α	TGCACTTTGGAGTGATCGGC	ACTCGGGGTTCGAGAGAAGATG
Human-IL-1β	CAGAAGTACCTAAGCTCGCCA	CTTGCTGTAGTGGTGGTCGG
Human-IL-6	TGCAATAACCACCCCTGACC	ATTTGCCGAAGAGCCCTCAG
Human-β-actin	CTCTTCCAGCCTTCCTTCCT	AGCACTGTGTTGGCGTACAG
Rat-AR	CGAGGCTGTGAAGGTTGCTA	TTGCTCCATAGCCGTCCAAG
Rat-TNF-α	CCAGGTTCTCTTCAAGGGACAA	CTCCTGGTATGAAATGGCAAATC
Rat-IL-1β	GCACAGTTCCCCAACTGGTA	AAGACACGGGTTCCATGGTG
Rat-IL-2	TCTGCAGCGTGTGTTGGATT	CTGGCTCATCATCGAATTGGC
Rat-IL-6	GTCAACTCCATCTGCCCTTCA	TGAAGTCTCCTCTCCGGACTT
Rat-IL-17A	TGAAGGCAGCGGTACTCATC	GGGTGAAGTGGAACGGTTGA
Rat-β-actin	AGCCATGTACGTAGCCATCC	CTCTCAGCTGTGGTGGTGAA

**Table 3:** Index of bone destruction

Radiological score	Bone erosion severity
0	Intact skeletal contours and normal joint gaps
1	Abnormality with any one or two metatarsals manifesting as minor bone erosion
2	Abnormalities in any 3-5 of bones presenting as bone erosion
3	Destructive abnormality with more than 5 bones with definite bone erosion;
4	Destructive abnormality with significant bone erosion of almost all metatarsals and complete erosion of at least one inner metatarsophalangeal joint, leaving some bony contour partially preserved.
