# Supplementary material for: N-Acetylcysteine overcomes epalrestat-mediated increase of toxic 4-hydroxy-2-nonenal and potentiates the anti-arthritic effect of epalrestat in AIA model
Source: Int J Biol Sci. 2023 Aug 6;19(13):4082–102. doi: 10.7150/ijbs.85028 (PMC10496504; doi:10.7150/ijbs.85028)
Supplement: Supplementary file 1 — Supplementary figure. [file ijbsv19p4082s1.pdf]

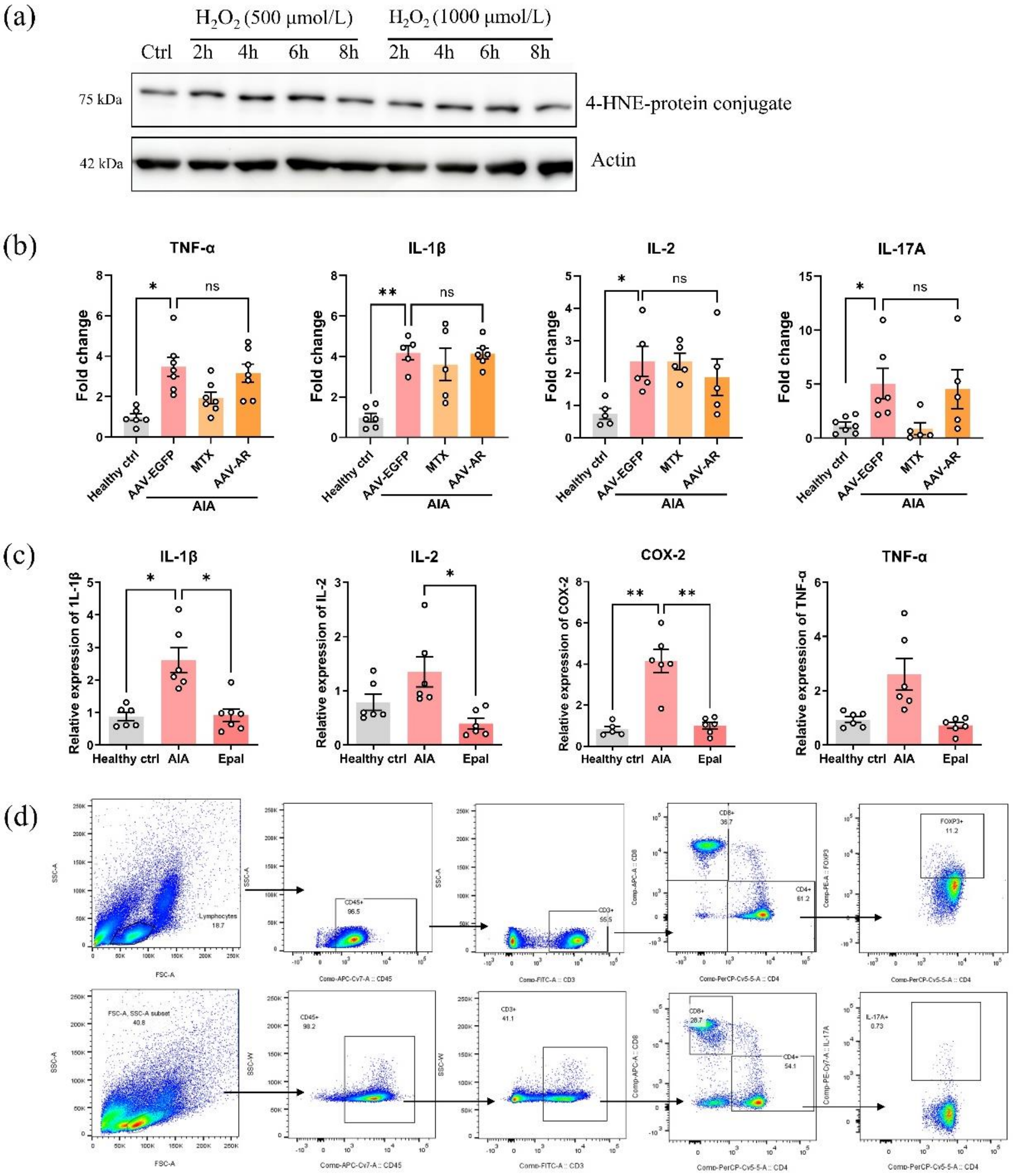

Figure. S1 (a) 500 or 1000 μmol/L H_2O_2 time-dependently induced the expression of 4-HNE-protein conjugate in RAFLS. Cells were treated with 500 or 1000 μmol/L H_2O_2 for indicated hrs. Cell lysates were analyzed by Western blotting using antibodies against 4-HNE-protein conjugate and β-actin (loading control). (b) Genes expression level of TNF-α, IL-1β, IL-2 and IL-17A in AAV-AR injected AIA group; (c) Genes expression level of IL-1β, IL-2, COX-2 and TNF-α in epalrestat-treated AIA group. Blood lymphocyte were obtained from different groups for RT-PCT. (d) Gating strategies for the analysis of Treg cells and Th 17 cells. The data shown are the means ± SEM ($n \geq 3$). *$P<0.05$, **$P<0.01$ vs healthy control group or AIA model group.
